# Test-and-treat approach to HIV/AIDS: a primer for mathematical modeling

**DOI:** 10.1186/s12976-017-0062-9

**Published:** 2017-09-05

**Authors:** Kyeongah Nah, Hiroshi Nishiura, Naho Tsuchiya, Xiaodan Sun, Yusuke Asai, Akifumi Imamura

**Affiliations:** 1 Graduate School of Medicine, Hokkaido University, Kita 15 Jo Nishi 7 Chome, Kita-ku, Sapporo, 060-8638 Japan; 2 CREST, Japan Science and Technology Agency, 4-1-8, Honcho, Kawaguchi-shi, Saitama, 332-0012 Japan; 3 Department of Preventive Medicine and Epidemiology, Tohoku Medical Megabank Organization, Tohoku University, 2-1 Seiryo-machi, Aoba-ku, Sendai, 980-8573 Japan; 4 Department of Applied Mathematics, Xi’an Jiaotong University, Xi’an, 710049 China; 5 Department of Infectious Diseases, Tokyo Metropolitan Cancer and Infectious Diseases Center Komagome Hospital, 3-18-22 Honkomagome, Bunkyo-ku, Tokyo, 113-8677 Japan

## Abstract

The public benefit of test-and-treat has induced a need to justify goodness for the public, and mathematical modeling studies have played a key role in designing and evaluating the test-and-treat strategy for controlling HIV/AIDS. Here we briefly and comprehensively review the essence of contemporary understanding of the test-and-treat policy through mathematical modeling approaches and identify key pitfalls that have been identified to date. While the decrease in HIV incidence is achieved with certain coverages of diagnosis, care and continued treatment, HIV prevalence is not necessarily decreased and sometimes the test-and-treat is accompanied by increased long-term cost of antiretroviral therapy (ART). To confront with the complexity of assessment on this policy, the elimination threshold or the effective reproduction number has been proposed for its use in determining the overall success to anticipate the eventual elimination. Since the publication of original model in 2009, key issues of test-and-treat modeling studies have been identified, including theoretical problems surrounding the sexual partnership network, heterogeneities in the transmission dynamics, and realistic issues of achieving and maintaining high treatment coverage in the most hard-to-reach populations. To explicitly design country-specific control policy, quantitative modeling approaches to each single setting with differing epidemiological context would require multi-disciplinary collaborations among clinicians, public health practitioners, laboratory technologists, epidemiologists and mathematical modelers.

## Background

Whereas the treatment of diseases has been conducted to expect its individual benefit, e.g. aiming for cure, it is vital to remember that the treatment of directly transmitted infectious diseases can also offer public benefits through indirect effect (e.g. decreased risk of infection due to decreased opportunity of secondary transmission and decreased cost for individuals other than those under treatment due to population impact of treatment). Such treatment for public interest is represented by the so-called “test and treat” approaches to HIV/AIDS [1]. Test-and-treat is an intervention strategy in which the population at risk is screened for HIV infection and diagnosed HIV infected individuals receive early treatment, aiming to eliminate HIV as it reduces the rate of spreading the virus to other people. The very first test-and-treat model by Granich and his colleagues has excellently resulted in conceptualizing a landmark of global health policy [2], inducing the world to be motivated to universally or at least radically screen HIV infected individuals in the population and promote their early treatment, not only for their own suppression from progression of HIV infection but also for the public benefit.

Nevertheless, the public benefit has also induced a need to justify goodness for the public, because nowadays “treatment as prevention” is no longer an individual interest but something to be ensured by the public or governmental organizations for its preventive performance [1]. The very first model of test-and-treat [2] has been repeatedly criticized for its practical utility, controversies and oversimplified model structure, and a number of alternative mathematical approaches have been proposed to assessing the population impact of test-and-treat strategy in both quantitative and qualitative manners. As it is valuable to overview mathematical approaches to test-and-treat strategy of HIV/AIDS for both general and expert readers, the present review article aims to briefly share the essence of contemporary understanding of the mathematical modelling of test and treat approaches as a primer.

## What is test-and-treat?

**Fig. 1:**
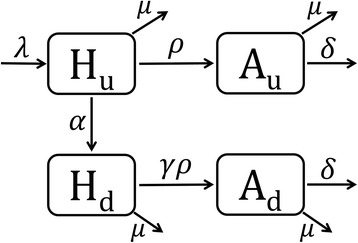
Flow chart of a simple compartmental model. Variable *H*_*u*_ [*H*_*d*_] is a fraction of undiagnosed [diagnosed] HIV-infected individuals without AIDS, *A*_*u*_ [*A*_*d*_] is a fraction of previously undiagnosed [diagnosed] AIDS cases

In the simplest manner, the test-and-treat strategy is mathematically captured by a four-compartmental model system (Fig. 1). While HIV infected individuals are at risk of developing AIDS in a matter of 10 years since infection, diagnosis of HIV in advance of AIDS could bring infected individuals under antiretroviral therapy (ART). Effective ART in preventing infected individuals from their pathophysiological progression to AIDS has been established to date and continuously improved over time [3]. In theoretical sense, ART at the population level is considered to offer three different types of impact, i.e., (i) reduced opportunity of secondary transmission [4, 5], (ii) reduced infectiousness per contact [6, 7], and (iii) individual impact including extended life expectancy [8] that reflects reduced risks of AIDS and AIDS death [3, 9]. Considering these benefits, Granich et al. [2] have shown that substantial herd immunity (or to be more precise “indirect population effect” of mass treatment; hereafter we use “herd immunity” for simplicity) could be attained by a combination of universal testing and expanded ART among all infected individuals. Assuming that a high adherence level is maintained for decades, it is anticipated that this policy helps to curb the HIV epidemic.

**Fig. 2:**
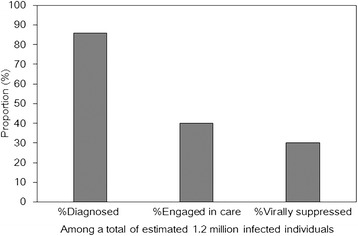
HIV care continuum in the United States, 2011. Estimated percentages of persons living with HIV infection are shown [11]. In 2011, an estimated 1.2 million persons were living with HIV infection in the United States

To attain such obvious indirect effect as induced by individual treatment series by HIV screening and treatment at a population level, it is essential to ensure that three key tasks are achieved, i.e., (i) finding HIV infected individuals, (ii) maintaining HIV care (i.e. retention to prevent drop-out) and monitoring CD4-positive T cell count and (iii) ensuring adherence and successful ART to suppress viral load. The Joint United Nations Programme on HIV/AIDS (UNAIDS) has introduced the concept of an HIV treatment cascade to identify and fill gaps in the continuum of services for testing, care and effective treatment. Following the 21st International AIDS Conference in Durban, South Africa, the UNAIDS report has led to a global slogan of “90–90-90” by 2020 that aims to achieve targets, which are that 90% of people living with HIV know their HIV infection, 90% of people who know their HIV infection are accessing treatment and 90% of people on treatment has enjoyed suppressed viral loads [10]. By the year 2030, UNAIDS is even aiming to achieve 95–95-95 at a global level. From a variety of countries, care cascade of the HIV/AIDS has been estimated and evaluated (e.g. Fig. 2 [11]), assisting respective country to point out the ongoing weakness of interventions. For instance, the case study of the United States in 2011 [11] indicates that the diagnostic coverage is close to reach 90%, while more than half of diagnosed individuals are not continuously engaged in care, and thus, their viral level is not brought under control by ART (Fig. 2). The critical point of the USA cascade in 2011 would thus be a need to ensure continued provision of care (i.e. improved retention) for diagnosed HIV infected individuals.

To date, a part of published empirical evidence indicated that widespread ART has led to reductions in nearly all epidemiological aspects of HIV/AIDS. For instance, expanded ART in Canada has been shown to be associated with decreased morbidity, mortality and HIV transmission, demonstrating that the combination of HIV testing and ART programs in Canada has had a promising and profound population impact [12]. On the other hand, while the reduced infectiousness has been shown to decrease HIV incidence, the ART certainly increases the life expectancy of people living with HIV/AIDS (PLWHA) and can sometimes increase the prevalence of HIV over time [13]. A more recent study has indicated that even the reduction in HIV incidence is not necessarily promised by test-and-treat program, especially if a part of 90–90-90 goal is not satisfied [14]. The importance of comprehensively understanding the pros and cons of test-and-treat strategy is increasingly recognized. Here we introduce a simple mathematical model, based on Fig. 1, to understand such controversy in the next section.

## Transmission dynamics of HIV under test-and-treat

Here we consider a simple mathematical model to understand how test-and-treat influences the population dynamics of HIV/AIDS in a rudimentary fashion. First, we divide the population into susceptible individuals, infected individuals without AIDS (*H*) and those who have been diagnosed as AIDS (*A*). Population *H* and *A* are further divided into undiagnosed (*H*_*u*_ and *A*_*u*_ *)* and diagnosed (*H*_*d*_ and *A*_*d*_) groups. Four compartments of HIV infected individuals have been schematically illustrated in Fig. 1. At least in this model, we assume that all diagnosed individuals are brought to be under ART.

Susceptible individuals experience infection with a rate *λ*(*t*) which is a function of infectious individuals *H*_*u*_ *, A*_*u*_, *H*_*d*_ and *A*_*d*_. We assume that ART reduces one’s infectiousness on a whole from *β* to ε*β* where parameter *ε* takes a value between zero and one, and the value 1 − ε represents the relative reduction in the transmissibility. Such reduction may not only be attributed to direct effectiveness of treatment (e.g. reduced viral load among infected individuals under treatment), but also caused by awareness of infection status and reduced frequency of risky sexual contact. Without treatment, infected individuals are assumed to develop AIDS with a progression rate *ρ*. HIV infected individuals under ART progresses to AIDS with a far smaller rate *γρ* where the value of 1 − *γ* would be between zero and one and $\frac{1}{\gamma \rho}-\frac{1}{\rho }$ scales the average gain of the extended time without AIDS. In addition to the natural death rate, *μ*, AIDS patients experience a higher mortality rate than HIV infected individuals, because of disease induced death rate *δ*. Parameter *α* represents the rate of diagnosis among HIV infected individuals, and 1/*α* gives the average waiting time for diagnosis.

The model is written as the system of ordinary differential equations.

$$ \frac{dH_u}{dt}=\lambda (t)\left(1-{H}_u(t)-{A}_u(t)-{H}_d(t)-{A}_d(t)\right)-\left(\alpha +\rho +\mu \right){H}_u(t), $$

$$ \frac{dA_u}{dt}=\rho {H}_u(t)-\left(\mu +\delta \right){A}_u(t), $$

$$ \frac{dH_d}{dt}=\alpha {H}_u(t)-\left(\gamma \rho +\mu \right){H}_d(t), $$

$$ \frac{dA_d}{dt}=\gamma \rho {H}_d(t)-\left(\mu +\delta \right){A}_d(t), $$

where the force of infection *λ*(*t*) is given by

$$ \lambda (t)=\beta {H}_u(t)+\varepsilon \beta {H}_d(t). $$

It should be noted that the transmission rate *β* reflects not only the infectiousness per contact but also the rate of sexual contact per unit time. To understand the concept of test-and-treat in the simplest manner, the model presented here has ignored gender and details of sexual partnership. Since AIDS patients are aware of their own infection status, we do not account for the infectiousness of AIDS patients for simplicity.

In the absence of diagnosis and treatment, the basic reproduction number, *R*_0_, the average number of secondary cases generated by a single primary case in a fully susceptible population, is given by linearizing the abovementioned system nearby the disease-free equilibrium, and we get

$$ {R}_0=\frac{\beta }{\rho +\mu }. $$

In the presence of diagnosis and treatment, the effective reproduction number, *R*_c_, the average number of secondary cases generated by a single primary case under test-and-treat policy is similarly derived as

$$ {R}_c=\frac{\beta }{\alpha +\rho +\mu }+\frac{\varepsilon \beta}{\gamma \rho +\mu}\frac{\alpha }{\alpha +\rho +\mu }. $$

Assuming that *ε* is almost negligible (i.e. zero), *R*_c_ is simplified as

$$ {R}_c=\frac{\beta }{\alpha +\rho +\mu }, $$

indicating that the reproduction number is reduced by a factor of *α* in the denominator as compared with *R*_0_. Early removal from (undiagnosed) infectious state plays a core role in characterizing the population impact of test-and-treat strategy.

To assess the test-and-treat strategy, a number of important and different epidemiological metrics have been quantified, e.g. common indicators include (i) the effective reproduction number, (ii) the incidence and prevalence given as the solution of the above mentioned system and (iii) the cost-effectiveness ratio as informed by the model outcome.

Different screening approaches would lead to different population outcomes. Such differing patterns of screening could arise in many ways, e.g. different frequency of HIV testing in the population, the use of advanced molecular techniques to detect those in the window period, targeted testing of high risk groups and different HIV infection stage (e.g. time since infection) to start treatment. Granich et al. [2] compared the cost of the so-called “opt-in” and “opt-out” strategies of testing. Opt-in strategy assumes that every infected individual presents to health services and starts ART at CD4+ count 350 cell/mL. Opt-out strategy assumes yearly universal voluntary testing of all individuals in the population, which is followed by immediate ART upon diagnosis of HIV infection. The study has shown that the cost of opt-in strategy will continue to increase whereas the cost of opt-out strategy would eventually decrease with a success of controlling HIV/AIDS at the population level.

**Fig. 3:**
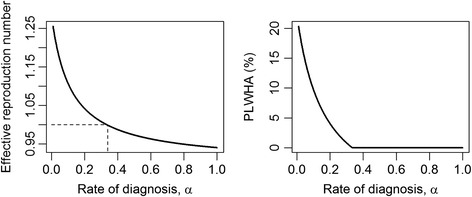
Test-and-treat with high screening rate may lead to the elimination of HIV. When the rate of diagnosis is greater than a certain threshold value, test-and-treat can successfully control HIV epidemic. Parameter values are μ = 1/60, ρ = 1/10, γ = 1/3, β = 0.15, δ = 1/2 and ε = 0.3

The suggested opt-out strategy is expected to eliminate HIV within 10 years and the reality on that point has been subject to extensive debate. Granich et al. [2] and Kretzschmar et al. [15] mathematically derived the elimination threshold and studied the conditions of treatment which makes the elimination of HIV feasible, such as the frequency of testing, test coverage or an initiation time of the ART. Figure 3 shows a simulation result of epidemic scenarios using the abovementioned equation system. Sensitivity of the effective reproduction number and PLWHA as a function of the rate of diagnosis *α* is examined. Given that the rate of diagnosis is greater than a certain threshold to lead to *R*_c_ < 1, the test-and-treat is proven to successfully control the HIV epidemic. The successful control endorses the global slogan of 90–90-90 strategy, targeting high enough diagnosis and treatment coverage to ensure substantial public benefit of HIV/AIDS.

**Fig. 4:**
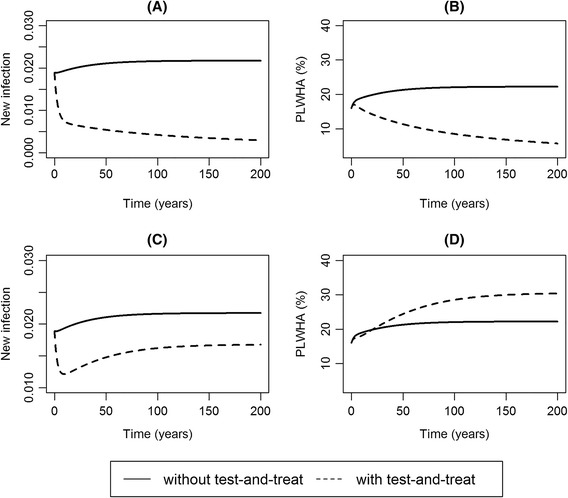
Test-and-treat could increase HIV prevalence. **a**, **c** The rate of change in HIV incidence, (**b**, **d**) the proportion of the PLWHA (people living with HIV/AIDS). Without test-and-treat policy, the rate of diagnosis was set as α = 0. Under the test-and-treat policy, α = 0.3 was adopted. Parameter values are μ = 1/60, ρ = 1/10, γ = 1/3, β = 0.15, δ = 1/2 and ε = 0.3. The test-and-treat reduces both the incidence and the prevalence in (**a**) and (**b**). For panel (**c**) and (**d**), ε = 0.5 was used instead of ε = 0.3 as the relative transmissibility for those who are diagnosed. In this scenario, test-and-treat increases HIV prevalence. Initial values are *H*_*u*_ = 0.15, *A*_*u*_ = 0.01, *H*_*d*_ = 0 and *A*_*d*_ = 0

Important pitfalls of test-and-treat are mainly seen in its long-term effects. For instance, the prevalence of HIV infection is not necessarily promised to decrease. Shafer et al. [16] estimated the population impact of ART in the future accounting for the change in the turnover rate of sexual partnership under ART. The model expected that ART will reduce the HIV incidence, while the HIV prevalence may be increased. Figure 4 compares two simple scenarios, i.e., long term dynamics with and without test-and-treat policy, comparing HIV incidence and prevalence. The numerical solutions are intentionally shown for the time-scale of 200 years (which is unrealistic!) in order that readers can recognize that the test-and-treat approach needs a patience to maintain high treatment coverage not merely for decades but sometimes for a very challenging long time.

Meeting certain mathematical conditions (especially, with a large value of *α* and very small *ε*), both HIV incidence and prevalence would decrease with time. Nevertheless, HIV prevalence in the presence of test-and-treat could exceed that without any control if the relative transmissibility of infected individuals under treatment is not sufficiently small. With the increased HIV prevalence, it follows that testing every year and immediate treatment upon diagnosis is not necessarily the most cost-efficient strategy and could even increase the long-term ART cost [17]. Theoretically, such controversial increase can be avoided by reducing the transmissibility for those who are diagnosed, for example, by ensuring high effectiveness of treatment, or by reducing the frequency of risky sexual contact after awareness of the infection state. Increase in HIV prevalence also indicates that the impact of test-and-treat should not be assessed by only a single epidemiological indicator, and multiple aspects of HIV epidemiology have to be carefully investigated, especially using the effective reproduction number or the elimination threshold.

In relation to the population impact, the HIV infection stage at the start of treatment has attracted researchers’ attentions [18], because the population impact of ART would be maximized if infected individuals are diagnosed at the very early stage of infection. In addition, at a late infection-age of HIV, the frequency of sexual contact is smaller than those in earlier stages [19].

## Future considerations

While many mathematical modeling studies exist, all have certainly agreed that increased diagnostic testing coupled with high retention of ART would induce a certain level of herd immunity to the population (e.g. [20]). Eaton et al. [21] systematically compared multiple models and found that published models substantially varied in their structure, complexity and parameter choices, but all suggest that high coverage and adherence to ART has the potential to reduce HIV incidence substantially. Mathematical modeling studies have found that model assumptions, especially many properties of the sex partner network, would have a profound impact on the incidence and prevalence, and incorporating local behavioral data is considered to be critical [17].

Due to the need to satisfy high diagnostic coverage and treatment, it is essential to first uncover the care cascade at each country level and locality. Depending on risk populations, the diagnostic coverage may greatly differ due to different awareness of risky behavior. A study in China has for instance focused on men having sex with men (MSM), demonstrating that HIV incidence is likely reduced by 50–70% subject to substantial scale up of diagnostic coverage and ART from 50 and 39% in 2010 to both 70% [22]. Understanding the transmission dynamics in the present day including the proportions of diagnosed, those followed-up and those adhered to HIV, the topical epidemiological question to answer may be to see if the effective reproduction number is achieved to be smaller than the value of one and if the elimination threshold was met. Country-specific case studies have to be conducted to confront with this task and understand the pros and cons with varying transmission dynamics by country. Depending on the epidemiological context and the coverages of cascade achieved, the optimal frequency of HIV testing is known to vary: opt-out strategy with HIV testing every year is not always the optimal policy choice [17].

Second, long-term epidemiological impact has yet to be explored in detail, preferably along with empirical datasets. In the presence of continued effort of test-and-treat approaches, HIV prevalence (or the number of PLWHA) and their life expectancy are expected to increase. These observations are likely to lead to ageing of infected individuals. Moreover, the aged infected individuals are more and more likely to experience chronic diseases including those not directly associated with HIV infection. Nevertheless, the failure to maintain high coverage of care and adherence to ART could lead to dramatic resurgence of the incidence and the surge of ART cost. Thus, dose adherence remains to be a key issue both in developing and industrialized countries (see [23] for resource limited settings). Not only in developing countries, but in the context of the United States with relatively low coverage of those engaged in care, Shah et al. [24] warned that failure to improve engagement in HIV care in the United States could lead to increase in HIV incidence, treatment cost and deaths, emphasizing the importance of retention in care. Another critical issue in the context of long-term impact is the emergence of drug resistant HIV with limited salvage regimen, unstable drug supply systems and the choice of first regimen (easily leading to cross-resistance) in resource-limited countries.

## Role of mathematical models in real settings

Not only leveraging the infrastructure and capacity for scaling up ART in resource limited settings, but the scale-up of diagnostic and treatment coverages of heterogeneous risk populations that are hard to reach are likely to be key issues in many practical settings [25]. Depending on epidemiological context of sexual mixing, transmission dynamics (incidence/prevalence) and heterogeneous risk groups, realistic quantitative approaches need to be sought supported by collaborations among clinicians, public health practitioners, epidemiologists and mathematical modelers. Collaborative ideas should also be extended to model-based analysis of data derived from trial studies with various designs [26].

For instance, heterogeneities in resource limited countries plays a key role not only in characterizing the non-homogeneous transmission dynamics of HIV but also in ensuring the validity of accessing to and maintaining high coverage among people most in need of ART. As mentioned in the context of the USA, the hard-to-reach population remains to be one of the biggest challenges even in more economically developed countries. Without accessing to the cluster of risky groups and ensuring their high treatment uptake, which are realistically very hard, the highly heterogeneous nature of sexual activity does not allow us to anticipate successful control of HIV/AIDS [27].

It is vital that modeling study results in different contexts (e.g. different locations such as sub-Saharan African countries vs middle-income countries, those focusing on opt-in and opt-out strategies of MSM population, and similarly those among commercial sex workers) preferably with empirical validations need to be more thoroughly shared, regardless of their success in reducing HIV incidence and prevalence. Such academic notions need to be accumulated and shared by highly variable epidemiological and economical settings.

## Conclusion

Mathematical modeling studies played a key role in designing and evaluating the test-and-treat strategy for controlling HIV/AIDS. This review article comprehensively discussed the essence of contemporary understanding of test-and-treat policy through mathematical modeling approaches and identified key pitfalls that have been identified to date. While the decrease in HIV incidence is achieved with certain coverages of diagnosis, care and continued treatment, HIV prevalence is not necessarily decreased and sometimes the test-and-treat is accompanied by increased long-term cost of antiretroviral therapy (ART). To explicitly design country-specific control policy, quantitative modeling approaches to each single setting with different epidemiological context area would require multi-disciplinary collaboration among experts of different disciplines.

## Abbreviations

AIDS: Acquired immunodeficiency syndrome
ART: Antiretroviral therapy
HIV: Human immunodeficiency virus
